# MODOMICS: a database of RNA modifications and related information. 2023 update

**DOI:** 10.1093/nar/gkad1083

**Published:** 2023-11-28

**Authors:** Andrea Cappannini, Angana Ray, Elżbieta Purta, Sunandan Mukherjee, Pietro Boccaletto, S Naeim Moafinejad, Antony Lechner, Charles Barchet, Bruno P Klaholz, Filip Stefaniak, Janusz M Bujnicki

**Affiliations:** Laboratory of Bioinformatics and Protein Engineering, International Institute of Molecular and Cell Biology in Warsaw, ul. Ks. Trojdena 4, PL-02-109 Warsaw, Poland; Laboratory of Bioinformatics and Protein Engineering, International Institute of Molecular and Cell Biology in Warsaw, ul. Ks. Trojdena 4, PL-02-109 Warsaw, Poland; Laboratory of Bioinformatics and Protein Engineering, International Institute of Molecular and Cell Biology in Warsaw, ul. Ks. Trojdena 4, PL-02-109 Warsaw, Poland; Laboratory of Bioinformatics and Protein Engineering, International Institute of Molecular and Cell Biology in Warsaw, ul. Ks. Trojdena 4, PL-02-109 Warsaw, Poland; Laboratory of Bioinformatics and Protein Engineering, International Institute of Molecular and Cell Biology in Warsaw, ul. Ks. Trojdena 4, PL-02-109 Warsaw, Poland; Laboratory of Bioinformatics and Protein Engineering, International Institute of Molecular and Cell Biology in Warsaw, ul. Ks. Trojdena 4, PL-02-109 Warsaw, Poland; Centre for Integrative Biology (CBI), Department of Integrated Structural Biology, IGBMC, 1 rue Laurent Fries, Illkirch, France; Centre National de la Recherche Scientifique (CNRS) UMR 7104, Illkirch, France; Institut National de la Santé et de la Recherche Médicale (Inserm) U964, Illkirch, France; Université de Strasbourg, Strasbourg, France; Centre for Integrative Biology (CBI), Department of Integrated Structural Biology, IGBMC, 1 rue Laurent Fries, Illkirch, France; Centre National de la Recherche Scientifique (CNRS) UMR 7104, Illkirch, France; Institut National de la Santé et de la Recherche Médicale (Inserm) U964, Illkirch, France; Université de Strasbourg, Strasbourg, France; Centre for Integrative Biology (CBI), Department of Integrated Structural Biology, IGBMC, 1 rue Laurent Fries, Illkirch, France; Centre National de la Recherche Scientifique (CNRS) UMR 7104, Illkirch, France; Institut National de la Santé et de la Recherche Médicale (Inserm) U964, Illkirch, France; Université de Strasbourg, Strasbourg, France; Laboratory of Bioinformatics and Protein Engineering, International Institute of Molecular and Cell Biology in Warsaw, ul. Ks. Trojdena 4, PL-02-109 Warsaw, Poland; Laboratory of Bioinformatics and Protein Engineering, International Institute of Molecular and Cell Biology in Warsaw, ul. Ks. Trojdena 4, PL-02-109 Warsaw, Poland

## Abstract

The MODOMICS database was updated with recent data and now includes new data types related to RNA modifications. Changes to the database include an expanded modification catalog, encompassing both natural and synthetic residues identified in RNA structures. This addition aids in representing RNA sequences from the RCSB PDB database more effectively. To manage the increased number of modifications, adjustments to the nomenclature system were made. Updates in the RNA sequences section include the addition of new sequences and the reintroduction of sequence alignments for tRNAs and rRNAs. The protein section was updated and connected to structures from the RCSB PDB database and predictions by AlphaFold. MODOMICS now includes a data annotation system, with ‘Evidence’ and ‘Estimated Reliability’ features, offering clarity on data support and accuracy. This system is open to all MODOMICS entries, enhancing the accuracy of RNA modification data representation. MODOMICS is available at https://iimcb.genesilico.pl/modomics/.

## Graphical Abstract

**Graphical Abstract:**
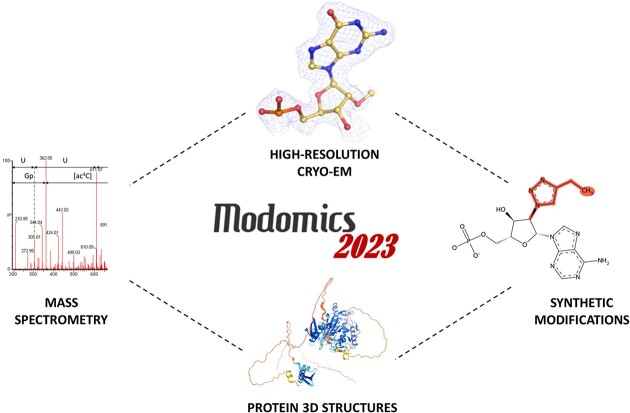

## Introduction

RNA modifications are chemical alterations to RNA nucleotides that have a profound impact on RNA structure and function. To date, >170 RNA modifications have been identified in all classes of RNA molecules. In living organisms, RNA modifications are introduced post- or co-transcriptionally by a variety of enzymes, and modification reactions can form complex pathways, leading to hypermodified residues. RNA molecules can also acquire modifications from external sources, such as the environment or other organisms (1). Moreover, specific RNA modifications are reversible, wherein the enzymatic removal of modified residues permits a rapid post-transcriptional adaptation to changing cellular or environmental contexts (2,3). RNA modifications have diverse effects on RNA molecules themselves, impacting their stability, structure, function, processing, and regulatory roles within the cell and they can be recognized by specialized reader proteins to fulfill their purpose. RNA modifications play important roles in all aspects of RNA metabolism, including RNA splicing, polyadenylation, transport, localization, translatability and stability (4). They can also be important for RNA interactions with other molecules, particularly proteins and ribonucleoproteins (5).

A growing area of interest lies in the deliberate introduction of artificial chemical modifications to RNA. These synthetic alterations are designed to bestow RNA molecules with functionalities that are not naturally occurring, serving specific experimental or therapeutic purposes (6,7). One prominent application of such modifications is in structural studies, where they provide enhanced stability to RNA molecules, ensuring that the RNA maintains its desired conformation during the analysis (8).

Over the past two years, the field of RNA modification has witnessed advancements in the novel technological approaches for detection and quantification of modified residues, in particular based on the nanopore technology (9–12). Advancements in cryo electron microscopy (cryo-EM) have significantly impacted RNA structure determination. Recent cryo-EM studies have achieved resolutions in the 2–3 Å range or better, enabling the identification of modified nucleotides for which the Coulomb density maps facilitate the direct visualization and atomic-level assignment of modified residues (13–16).

## Database content

**Figure 1.**
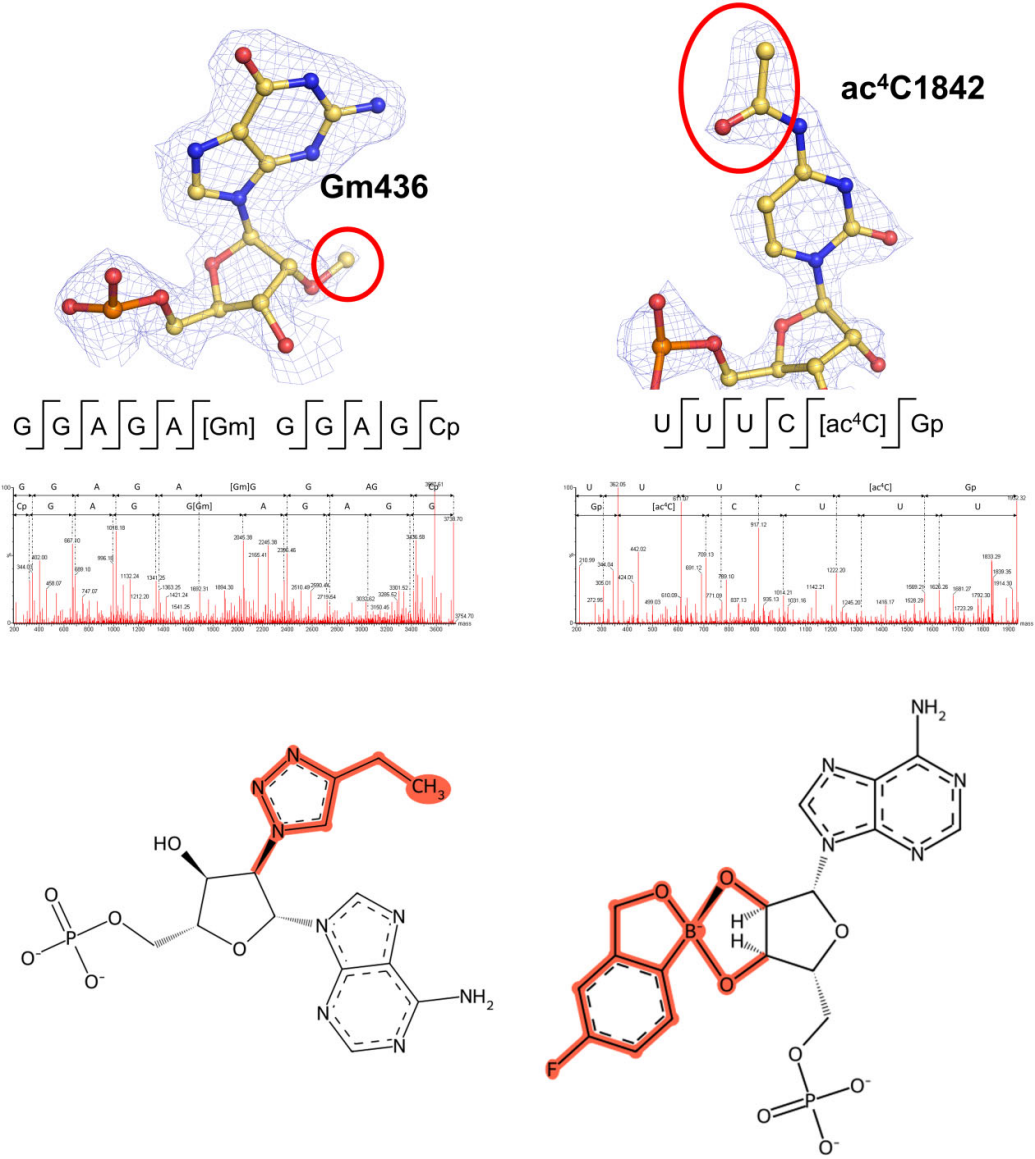
Illustration of the key new features in MODOMICS. Top panel: Examples of modifications sites identified in rRNAs using high-resolution cryo-EM and fragment-based MS analysis of 80S ribosomes. The modified ribonucleotides are shown in the context of the Coulomb density map displayed as blue mesh, with modifications indicated by red ellipsoids. Below, the validation by mass spectrometry using enzyme-induced RNA fragment analysis is illustrated. Bottom panel: 2D schemes of ribonucleotide residues imported to MODOMICS: 2′-deoxy-2′-(4-ethyl-1H-1,2,3-triazol-1-yl)adenosine 5′-(dihydrogen phosphate), left; [(6-amino-9H-purin-9-yl)-[5-fluoro-1,3-dihydro-1-hydroxy-2,1-benzoxaborole]-4′yl]methyl dihydrogen phosphate (ANZ), right. Chemical modifications are highlighted in red.

As in the previous release (17), MODOMICS hosts a catalog of modified residues, enzymes and guide RNAs responsible for individual reactions, RNA modification pathways, sequences of modified RNAs, a catalog of ‘building blocks’ for chemical synthesis of modified RNA, links of RNA modifications to different diseases, and other associated data such as relevant publications. The updated MODOMICS introduces new data types, including a coding system that expands the catalog of modified residues to include synthetic molecules, and an annotation system that indicates the nature and reliability of the data interpretation (Figure 1).

### Updated modifications section

The catalog of modifications in MODOMICS includes ribonucleosides and ribonucleotides present in RNAs existing in nature, which are introduced into RNA post- or co-transcriptionally by various enzymes. In the previous release (17), we added sequences of experimentally determined RNA structures that contained modifications present in nature. However, some of these RNAs also contained deoxyribonucleotide residues, and various chemically synthesized residues that had no counterpart among the naturally present modifications, e.g. locked nucleic acids (LNAs), phosphorothioate-modified nucleic acids, fluorine substitutions, and many others, which weren’t included in MODOMICS. For the current release, the catalog of modified ribonucleotide residues was expanded to include 79 modified residues found in experimentally determined RNA structures, including ‘unnatural’ ones (not found in nature). Currently, MODOMICS enables the inclusion of modified residues that are not a part of natural modification pathways. This expansion improved the correspondence between MODOMICS and the entries in the RCSB Protein Data Bank, covering both natural and synthetic residues (18).

The extension of MODOMICS to handle potentially a very large number of synthetic residues required an update of the nomenclature system, which was originally developed to deal with only a limited number (e.g. a few hundred) of naturally existing nucleoside and nucleotide residues. Apart from full chemical names, MODOMICS uses several codes to denote modified residues, including:

- short names, traditionally used in literature, e.g. Am, m^1^A, io^6^A, cm^5^s^2^U, pms^2^i^6^A etc.
- a single character code (19), this system is particularly useful for the presentation of sequence alignments, where each column must contain only a single character.
- MODOMICS code, introduced in the 2017 update (20) that uses one or more digits and one letter (A, G, C, U) as a unique identifier to represent each modified residue. It was designed to be readable both for humans and computer software. For example, modified residues Am, m^1^A, io^6^A, cm^5^s^2^U, pms^2^i^6^A were indicated as 0A, 1A, 60A, 2540U and 2161551A, respectively.

In this update of MODOMICS, modifications had to be introduced into all three codes. For short names of unnatural residues that are represented in the RCSB database, MODOMICS uses the RCSB LIG codes, e.g. 5BU for 5-bromo-uridine-5′-monophosphate, LCC for [(1R,3R,4R,7S)-7-hydroxy-3-(5-methylcytosin-1-yl)-2,5-dioxabicyclo[2.2.1]hept-1-yl]methyl dihydrogen phosphate. For natural residues, MODOMICS continues to use traditional names, regardless of whether they have RCSB LIG codes or not. For unnatural residues not available in the RCSB PDB that may be included in MODOMICS in the future, relevant short names will be provided. The single character code was extended to Unicode UTF-8 characters, including those from the Chinese alphabet. We also replaced some of the previously used characters, which commonly caused problems for computational parsing or displaying plain text files (such as ‘, ’, “, ”, or =). MODOMICS presents both the previous (now obsolete) and the new updated, computer-friendly single-character code.

The MODOMICS code was subjected to a major overhaul. To handle multiple unnatural residues, we extended it to use uniformly 10 digits and a letter. To keep the code both human- and computer-friendly, the first digit serves as a barcode to indicate the type of sugar moiety in nucleoside and nucleotide residues i.e. D-deoxyribose, d-ribose, or another (‘unnatural’) moiety, and the presence of a regular phosphate group or a modified phosphate group in nucleotide residues (see Table 1 for examples and explanation). The letter in the code is A, U, C or G if the base moiety is: (i) an unmodified adenine, uracil, cytosine, or guanine (regardless of the biosynthetic pathway, e.g. a result of C to U editing is simply U); (ii) a chemically modified (naturally or unnaturally) derivative of adenine, uracil, cytosine or guanine that keeps all the non-hydrogen atoms of A, U, C or G; (iii) if it is a result of a naturally occurring chemical modification of adenine, uracil, cytosine or guanine, even if non-hydrogen atoms of the respective natural unmodified base are not maintained after the modification (e.g. G for queuosine, A for inosine). In all remaining cases, in particular for residues that do not possess all the non-hydrogen atoms of A, U, C or G (e.g. for 2-amino-9-[2-deoxyribofuranosyl]-9H-purine-5′-monophosphate or d-ribofuranosyl-benzene-5′-monophosphate), the letter is X. The entire string of 10 digits and a letter is a unique identifier for a chemical moiety entry in MODOMICS.

**Table 1.** Examples of residues and their new MODOMICS codes

Moiety name	Short name	MODOMICS code	Phosphate	Sugar	Base
1-methyladenosine	m^1^A	2000000001A (old code 1A)	absent	ribose	A modified (natural)
N(1)-methyladenosine 5′-monophosphate	pm^1^A	2000001551A (old code 1551A)	natural	ribose	A modified (natural)
5-bromo-2′-deoxy-cytidine-5′-monophosphate	CBR	1000000030C	natural	deoxyribose	C modified (unnatural)
2-amino-9-[2-deoxyribofuranosyl]-9H-purine-5′-monophosphate	2PR	1000000005X	natural	deoxyribose	other (unnatural)
5-bromo-uridine-5′-monophosphate	5BU	2000000010U	natural	ribose	U modified (unnatural)
purine riboside-5′-monophosphate	P5P	2000000056X	natural	ribose	other (unnatural)
isoguanosine-5′-monophosphate	IG	2000000043A	natural	ribose	A modified (unnatural)
uracil arabinose-5′-phosphate	UAR	3000000069U	natural	unnatural	U (natural)
3′-amino-3′-deoxyadenosine 5′-(dihydrogen phosphate)	8AN	3000000015A	natural	unnatural	A (natural)
guanosine-5′-thio-monophosphate	GS	4000000041G	unnatural	deoxyribose	G (natural)
adenosine-5′-(dithio)phosphate	ADS	5000000022A	unnatural	ribose	A (natural)
uridine 5′-monothiophosphate	U37	5000000065U	unnatural	ribose	U (natural)

Barcode (first digit) 1 = sugar is d-deoxyribose or its derivative (retains all non-hydrogen atoms of d-deoxyribose) and phosphate is natural (in nucleotides) or absent (in nucleosides); 2 = sugar is d-ribose or its derivative and phosphate is natural or absent; 3 = sugar is unnatural and phosphate is natural or absent. 4 = sugar is d-deoxyribose or its derivative, and phosphate is unnatural; 5 = sugar is d-ribose or its derivative, and phosphate is unnatural; 6 = sugar is unnatural and phosphate is unnatural (such cases are not yet present in MODOMICS).

All the new chemical moieties imported to MODOMICS were checked by experts in our team. This process included the standardization of structures and correcting/adding stereochemical information. As for the moieties previously available, various physicochemical properties were calculated. Each chemical structure is available for download in multiple formats.

### Updated RNA sequence section

The RNA sequences section in MODOMICS was updated. From RCSB we imported 900 new sequences with naturally/unnaturally modified residues and updated two existing entries previously imported to introduce such residues absent in earlier MODOMICS versions. We added 15 tRNA sequences (21) and 15 rRNA sequences (22). Users can download individual sequences in the FASTA format, with modifications represented as the MODOMICS code, single character code, or short names.

### Alignments of selected families of RNA sequences with modifications

The MODOMICS database used to have a section with sequence alignments of tRNAs and rRNAs (LSU and SSU). It was removed during one of the previous updates when a new relational data model for handling sequences was introduced. We now restored this section. ‘Unmodified’ versions of sequences from MODOMICS were aligned to covariance models from Rfam (23) using Infernal (24). They were then replaced with the modified versions and the alignments were curated manually, taking the 3D structure information into account. New functions were added for data retrieval. Users can download the alignments in the multiple-sequence gapped FASTA format (which is relevant only when modifications are represented in a single Unicode character code), or in a tab-separated values (tsv) format, which enables the presentation of modified residues in various formats, without breaking the correspondences in columns.

### Updated collection of proteins involved in RNA modification

The MODOMICS section on proteins involved in RNA modification pathways was updated to add 57 proteins involved in RNA editing. Annotations of various protein entries were updated based on the literature review. We linked MODOMICS protein entries to over 1000 experimentally verified structures of proteins involved in RNA modification from the RCSB PDB database (including multiple RCSB entries for a number of individual proteins). Whenever available, we linked the MODOMICS entries to 3D structural predictions from the AlphaFold database (25). The interface was updated to enable visualization of structures and downloading the atomic coordinates or exporting their sequences. Protein sequences can be downloaded in FASTA format.

### Annotation of data in MODOMICS

We enhanced the data structure to facilitate more detailed annotations. Specifically, we introduced two annotation features: evidence indicates the level of experimental or computational support for a given data feature, along with its source (e.g. specific publication, database import, method used, computational prediction or human inference from metadata). Estimated reliability represents the likelihood of the data's accuracy based on the available evidence.

For evidence, we defined six classes: (i) multiple direct experimental evidence (validated using at least two different direct methods), (ii) direct experimental evidence (characterized by one direct method); (iii) inferred based on indirect experimental evidence; (iv) predicted computationally; (v) unknown (evidence not yet annotated); (vi) irrelevant (evidence cannot be estimated for a given data type). For estimated reliability, we defined six classes as well: (i) highly reliable; (ii) solid (large errors are unlikely, but cannot be completely excluded, minor errors should not undermine the meaning of the data); (iii) speculative (errors expected); (iv) questionable (e.g. conflicting reports in the literature and in databases); (v) unknown (assessment not yet performed); (vi) irrelevant (cannot be estimated for a data type). This annotation system applies to all MODOMICS entries, encompassing data obtained experimentally, computationally, or both. Examples include:

- Presence of a modified residue in a specific RNA position. For instance, if identified by mass spectrometry and visualized in a high-resolution cryo-EM Coulomb density map, it would be classified as evidence class 1 and reliability class 1. Inference from cDNA sequencing mutation patterns would be evidence class 3 and reliability class 3.
- Determining a protein's role in a modification reaction. An *in vitro* activity without *in vivo* confirmation would be evidence class 2 and reliability class 2. Computational prediction based on similarity to a characterized homolog would be evidence class 4 and reliability class 3.
- Associating an RNA-modifying enzyme with a disease. Correlation of expression patterns with disease symptoms, lacking a mechanistic explanation, would be evidence class 3 and reliability class 3.
- Computational alignment of RNA family sequences. Alignments for extant sequences, being computational, would be evidence class 4. Manually curated alignments would typically be reliability class 2, while automated alignments of divergent sequences would be class 3.

In this update, we primarily focused our annotation efforts on modifications in rRNA and tRNA sequences. Our goal is to systematically annotate all RNA types and data categories in the upcoming future. At present, any unannotated data is labeled under evidence and reliability class 5, which signifies pending annotation and reliability assessment. As always, MODOMICS users are encouraged to provide feedback to enhance the annotations.

### Future prospects

In upcoming MODOMICS updates, we plan to include the annotation of prevalence for individual positions of modified residues. This will detail the frequency of specific features at given positions in RNA molecules, shedding light on modifications present in only a fraction of an RNA population. We also plan to expand the collection of synthetic modifications, including ones beyond those entries in RCSB. We plan to cover new therapeutic strategies that are being developed to target RNA modifications, in particular small molecule inhibitors of RNA modification enzymes and RNA-modifying proteins. We will continue cross-referencing with other databases to ensure comprehensive coverage of RNA modification data. Enhancements to the user interface are also planned to streamline data access, visualization and feedback.

## Data Availability

The data are accessible freely for research purposes at https://iimcb.genesilico.pl/modomics/.
